# Shadow Detection in Still Road Images Using Chrominance Properties of Shadows and Spectral Power Distribution of the Illumination

**DOI:** 10.3390/s20041012

**Published:** 2020-02-13

**Authors:** Manuel José Ibarra-Arenado, Tardi Tjahjadi, Juan Pérez-Oria

**Affiliations:** 1 Department of Electrical and Energy Engineering, University of Cantabria, Avda. Los Castros s/n, 39005 Santander, Spain; 2 School of Engineering, University of Warwick, Gibbet Hill Road, Coventry CV4 7AL, UK; t.tjahjadi@warwick.ac.uk; 3 Department of Electronic Technology and Automatic Systems, University of Cantabria, Avda. Los Castros s/n, 39005 Santander, Spain; juan.perezoria@unican.es

**Keywords:** advanced driving assistance systems, illumination, shadow detection, shadow edge, road detection

## Abstract

A well-known challenge in vision-based driver assistance systems is cast shadows on the road, which makes fundamental tasks such as road and lane detections difficult. In as much as shadow detection relies on shadow features, in this paper, we propose a set of new chrominance properties of shadows based on the skylight and sunlight contributions to the road surface chromaticity. Six constraints on shadow and non-shadowed regions are derived from these properties. The chrominance properties and the associated constraints are used as shadow features in an effective shadow detection method intended to be integrated on an onboard road detection system where the identification of cast shadows on the road is a determinant stage. Onboard systems deal with still outdoor images; thus, the approach focuses on distinguishing shadow boundaries from material changes by considering two illumination sources: sky and sun. A non-shadowed road region is illuminated by both skylight and sunlight, whereas a shadowed one is illuminated by skylight only; thus, their chromaticity varies. The shadow edge detection strategy consists of the identification of image edges separating shadowed and non-shadowed road regions. The classification is achieved by verifying whether the pixel chrominance values of regions on both sides of the image edges satisfy the six constraints. Experiments on real traffic scenes demonstrated the effectiveness of our shadow detection system in detecting shadow edges on the road and material-change edges, outperforming previous shadow detection methods based on physical features, and showing the high potential of the new chrominance properties.

## 1. Introduction

**Figure 1:**
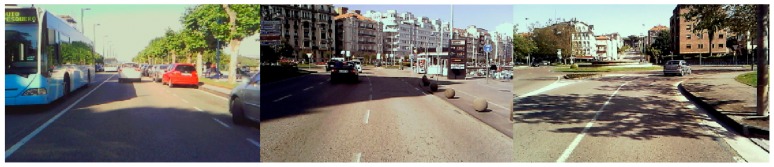
The presence of shadows entails a difficult challenge in vision-based road detection systems.

Increasingly powerful computers and advances in the fields of image processing and computer vision make vision-based systems one of the fastest growing segments in advanced driver assistance systems (ADAS). There are several factors that make onboard systems based on computer vision challenging. Changing scenarios, cluttered backgrounds, variable illumination, and the presence of objects of different class in the scene contribute to making the design of driver assistance tasks such as the detection of roads [1,2] and lanes [3,4] difficult. One of the most challenging factors encountered by a vision-based ADAS system is cast shadows [1,5] (see Figure 1). Shadows on a road may cause apparent merging of objects in road scenes captured by a video camera, as well as alterations in the shape and color of objects and road, which result in poor region segmentation. As a consequence, shadowed road regions can easily be misclassified as objects instead of road, which may lead to system error. Motivated by the undesirable effect of shadows, this paper presents a set of new physical properties to better characterize shadows on the road so as to minimize the possible misclassification of non-shadowed road regions, and objects as shadows. We use the new properties to design a shadow edge detection method intended to integrate a complete onboard road detection system which mainly consists of the classification of image pixels as belonging or not to the road surface [1].

The identification of cast shadows is not only important in vision-based ADAS systems but in general applications; thus, it is extensively studied [6,7,8,9]. Existing shadow detection approaches can be classified into two main categories [6]: model-based and property-based methods. The former is highly dependent on the environment, taking into account a priori scene information such as light source direction and geometry of the objects [10]. They are, thus, not applicable for onboard systems where no assumptions of the scene can be made. Property-based methods on the other hand, are more suitable for general applications. They are based on comparing the pixel properties of a candidate shadow region and those of a non-shadowed reference region of the same material surface. In static background applications consisting of a video sequence captured by a fixed camera [11,12,13,14,15,16], moving shadows are detected using background subtraction techniques [17,18,19] and comparing properties of pixels in the current frame of the sequence to background pixels in the reference frame devoid of shadows. However, such a technique is not effective for ADAS, since the road scene is continuously changing. Instead, two alternative strategies are applicable to onboard systems. One strategy focuses on comparing pixel properties between the candidate shadow region and a selected region located at the bottom of the image, which is assumed a free road region in front of the ego-vehicle [1,4,20]. However, depending on the distance to the camera, the varying reflection angles of the illumination may cause color variation of the road; thus, even a well-laid asphalted road can show zones in the image where the pixel properties are significantly different. This fact may lead to shadow misclassification when the candidate shadow region is far from the bottom of the image. The second strategy exploits locality and focuses on the comparison of pixel properties of regions across image edges [6,21,22], where the region on the darker side of an edge is a candidate shadow region and the region on the brighter side is assumed the non-shadowed reference region. As a result, an image edge is classified as an edge due to a shadow boundary or a material change. Once a shadow edge is identified, the image region on the darker side of the edge is assumed the shadow.

In order to compare the pixel properties, property-based methods rely on shadow features such as texture [23], gradients [24], histograms [25], and spectral composition [6,11,13], including luminance and chrominance. The use of spectral composition is mainly based on the assumption that shadows reduce the surface brightness without significantly modifying its chromaticity. This observation is effective for applications where the spectral power distribution of the illumination (SPD) is similar for both shadowed and non-shadowed regions; thus, the surface color components vary linearly. Approaches based on this consideration are known as color-invariant methods and they are widely used in ADAS applications exploiting different color spaces such as red–green–blue (RGB) [26,27], normalized RGB [28], Hue–Saturation–Intensity (HSI) [29,30], Hue–Saturation–Value (HSV) [11,16,31], Improved–Hue–Saturation–Luminance (IHSL) [32], YUV [33,34,35], c1c2c3 [6], and l1l2l3 [36]. However, in outdoor scenes, the illumination is composed of sunlight and skylight, which have different SPDs. Non-shadowed regions are illuminated by daylight (i.e., skylight and sunlight), whereas shadowed regions are only illuminated by skylight; thus, their chromaticity varies. In order to address this issue, methods based on physical properties consider the SPD of the illumination and the surface reflectivity properties.

Early physics-based approaches were based on the observation that the intensity of each red, green, and blue (RGB) component of a surface decreases across a shadow edge [37]. This shadow feature is used by practically all methods. The bluish effect of shadows was also widely exploited. The fact that shadows are only illuminated by skylight (predominantly blue) makes the normalized blue component of a shadowed region greater than in a non-shadowed one [38]. In References [29,39], it was assumed that the blue component of shadows was dominant over the red and green. However, this assumption is not always true, since surfaces with a strong dominance of red or green may maintain their dominance when shadowed. In Reference [40], it was also observed that the red component of the sunlight was dominant; thus, the normalized red component of a shadowed region decreased. This observation is generally satisfied in the umbra of shadow but not in the penumbra, owing to it being illuminated by some sunlight. More recently, the method in Reference [12] exploited the fact that the intensity change across a shadow edge was greater in the red and green components than in the blue, whereas the method in Reference [41] presented a set of relationships between the attenuation of each RGB channel across a shadow edge and the RGB values of the non-shadowed region. The former is applicable only on low-saturated surfaces whereas the latter assumes the SPDs of skylight and sunlight as constant, which is not true, since they vary significantly during the day. A well-known shadow feature was presented in Reference [42] where shadows were identified using color ratios across image edges. It assumed that the color ratios across boundaries of shadows cast onto the different surfaces were similar since they were due to the same illumination change. Although these ratios may fail in complex real images [42], several approaches were built upon them [20,22,43,44].

A different physics-based approach to detect shadows is based on illumination invariance. In Reference [45], shadow boundaries were detected by comparing edges in the input RGB image to edges found in the one-dimensional illumination-invariant shadow-free image obtained by the color-constancy method in Reference [46]. Despite the fact that this method is not reliable in images where shadow edges are not well defined [45], it was widely exploited [1,47,48,49,50]. However, most of these illumination-invariant methods require user intervention, as well as high-quality images with wide dynamic range and calibrated sensors, failing severely with consumer-quality images [44].

Generally, in order to improve robustness, most physics-based methods combine more than one shadow feature [6,12,15,44,51,52]. Currently, some of them address the shadow detection by learning techniques [13,16,21,44,51,53,54,55,56]. In Reference [21], support vector machines (SVM) were trained using color ratios to identify shadow edges in typical images. In Reference [51], an SVM classifier was trained using intensity ratio, chromatic alignment, and both color and texture histograms. A conditional random field classifier trained using color ratios and texture information was proposed in Reference [44]. This method focused on detecting shadows on the ground in consumer-quality photographs. In Reference [13], the Gaussian mixture model (GMM) was used to learn the properties of shadowed background surfaces to detect moving cast shadows. In Reference [56], two convolutional neural networks (CNNs) were combined to learn features of regions inside the shadow (umbra) and regions adjacent to the shadow boundaries (penumbra), since both shadowed regions presented different types of features. However, although learning-based methods demonstrated high robustness in specific scenarios, they are likely to fail in images slightly different to those used for their training [22].

Despite the numerous methods, shadow detection remains a very challenging task, since shadow features may be shared by objects whose chrominance features are unpredictable. Therefore, new properties are important to better characterize shadows, minimizing the misclassification of objects and non-shadowed regions.

The contributions of this paper are manifold. We firstly derive and validate the following set of new chrominance properties of shadows based on the Planckian illumination and Lambertian surface model, as well as the SPD of the illumination, to effectively characterize shadows on road:

**Property 1.** The relationship between the red and green surface reflectances due to sunlight is higher or equal to that due to skylight.

**Property 2.** The red component of the road reflectance due to sunlight is dominant, being higher than the blue, and higher or equal to the green one. In the same way, the green component of the road reflectance is higher than the blue one.

**Property 3.** The change in the red–green proportion of the road reflectance due to skylight and sunlight is smaller than the change in the red–blue one. This observation is also valid when comparing the changes of the surface relationships green–red and green–blue.

Associated with Property 1, we propose one constraint between the red and green surface reflectances due to sunlight and skylight. Associated with Property 2, we propose three constraints to consider the effect of sunlight on neutral surfaces. Associated with Property 3, we propose two constraints to take into account both the similarity of the red and green components of the illumination and the large variation of the blue component. These chrominance properties and constraints are utilized as shadow features in a shadow edge detection algorithm as a preprocessing stage intended to integrate a complete onboard road detection system for driver assistance. Since onboard systems deal with still images, there is not a known non-shadowed reference road region in the image to compare the pixel properties. Thus, the method focuses on detecting shadow boundaries by comparing pixel properties across image edges. No prior knowledge of the scene, camera calibration, or spatio-temporal restrictions are required, and static background applications can also be addressed. The method identifies image edges delimiting shadows and non-shadowed road regions by verifying whether the pixel values of regions on both sides of the edge under analysis satisfy the new constraints imposed.

The remainder of this paper is organized as follows: Section 2 presents the reflection model, as well as discusses the SPDs of skylight and sunlight, including their effects on shadowed and non-shadowed asphalt road surface. Section 3 presents the new chrominance properties of shadows, and Section 4 describes the proposed shadow edge detection method. Experimental results are shown and discussed in Section 5. Finally, Section 6 concludes the paper.

## 2. Physics Basis: Reflection Model and SPD of the Illumination

### 2.1. Reflection Model

Assuming a Planckian illumination and Lambertian surface model as in References [6,22,45,57], the light reflected off a point *p* on a surface is the product of the SPD of the incident illumination *E*(*λ*, *p*) and the surface reflectance *S*(*λ*, *p*). Thus, for some illumination and viewing geometry, the response of a digital camera sensor *C_i_* at a given image pixel (*x*, *y*), which corresponds to a surface point *p* of the scene, can be expressed as in References [6,21,22,45,46,57],

(1) $$C_{i}(x,y)=\int_{w}E(\lambda ,x,y)\times S\left(\lambda ,x,y\right)\times Q_{i}(\lambda )\times d\lambda ,$$

where *C_i_* ϵ {*R*, *G*, *B*} are the red, green, and blue sensor responses, *λ* is the wavelength, *w* is the visible spectrum range, and *Q_i_*(*λ*) ϵ {*Q_R_*(*λ*), *Q_G_*(*λ*), *Q_B_*(*λ*)} are the spectral sensitivities of the three color camera sensors. Assuming camera filters of infinitely narrow bandwidth as in References [11,21,22,40,42,45,58], it is possible to represent them by impulse functions which are centered on the filter’s characteristics (e.g., Dirac delta function: *Q_i_*(*λ*) = *q_i_*⋅*δ*(*λ* − *λ_i_*) [21,22,45]). With this approximation, Equation (1) becomes

(2) $$C_{i}(x,y)=E(\lambda_{i},x,y)\times S\left(\lambda_{i},x,y\right)\times q_{i},$$

where *λ_i_* is the center frequency of the *i-*th channel filter, and *q_i_*ϵ{*q_R_*, *q_G_*, *q_B_*} are spectral sensitivity factors of the three color camera sensors. Equation (2) represents the three color components of the reflected light due to a single illumination source. However, in outdoor scenes, the illumination is due to the contribution of two illumination sources, sunlight (*E_sun_*(*λ_i_*)) and skylight (*E_sky_*(*λ_i_*)), with different SPDs. In line with References [6,12,41,47], the inter-reflections due to nearby objects can be disregarded, since the energy of inter-reflection decays exponentially for each reflection [12]. Therefore, the sensor measurement for an image pixel (*x*, *y*) corresponding to a non-shadowed surface point of the scene is

(3) $$C_{i}{\left(x,y\right)}_{non-sha}=\left[E_{sky}\left(\lambda_{i},x,y\right)+E_{sun}\left(\lambda_{i},x,y\right)\right]\times S\left(\lambda_{i},x,y\right)\times q_{i},$$

giving a three-dimensional (3D) color vector *C_i_*(*x*, *y*)*_non-sha_* = [*R_non-sha_*, *G_non-sha_*, *B_non-sha_*]. The response for a pixel in the shade is obtained from Equation (3) making *E_sun_*(*λ_i_*, *x*, *y*) = 0, i.e.,

(4) $$C_{i}{\left(x,y\right)}_{sha}=E_{sky}\left(\lambda_{i},x,y\right)\times S\left(\lambda_{i},x,y\right)\times q_{i},$$

giving a color vector *C_i_*(*x*, *y*)*_sha_* = [*R_sha_*, *G_sha_*, *B_sha_*].

### 2.2. SPD of the Illumination in Outdoor Scenes

The sun emits white light, which penetrates the atmosphere and is scattered in all directions by gas molecules in the air. However, due to the small size of the molecules, the scattering (Rayleigh scattering) is more effective at short wavelengths which correspond to blue, thus giving the sky a bluish color. On the other hand, most of the light comprising the remaining wavelengths (from green to red) passes through the atmosphere and reaches the earth surface. The mixture of red and green produces a yellowish sunlight, which may attain a reddish tone at certain hours of the day.

#### 2.2.1. SPD of Skylight

A shadow on a road appears when an object occludes the sunlight; thus, only the bluish skylight illuminates the road. Although the intensity of the skylight can vary depending on the time of day and atmospheric conditions, during most parts of the day, the red and green components (*E_sky_*(*λ_R_*), *E_sky_*(*λ_G_*)) present similar values, combining to give a higher blue component (*E_sky_*(*λ_B_*)). However, as the sun gets lower in the sky, the sunlight passes through more of the atmosphere, which produces an increase in the scattering of its green wavelength. Hence, the green component of the skylight increases relative to red. This intensity difference between the red and green components is generally small and does not cause a significant change in the appearance of the sky (a greenish skylight is not usual). Thus, depending on the time of day when skylight illuminates a road, the green component of the light reflected from the road surface can be considered to be affected by a similar or a slightly higher quantity than the red, whereas the blue is affected by a larger quantity, i.e.,

(5) $$\begin{array}{c} \;E_{sky}\left(\lambda_{R}\right)\leq E_{sky}\left(\lambda_{G}\right),\\ \;E_{sky}\left(\lambda_{R}\right)<E_{sky}\left(\lambda_{B}\right),\\ E_{sky}\left(\lambda_{G}\right)<E_{sky}\left(\lambda_{B}\right), \end{array}$$

#### 2.2.2. SPD of Sunlight

A non-shadowed road region is illuminated by both skylight and sunlight. During most part of the day, the sunlight is yellowish since its red and green components (*E_sun_*(*λ_R_*), *E_sun_*(*λ_G_*)) remain very similar with respect to each other, combining to give a smaller blue component (*E_sun_*(*λ_B_*)). The red–green equilibrium is attained at noon. As the sun gets lower in the sky, the green component of the sunlight decreases relative to red, making the sunlight orangish until sunset when it may attain reddish. From sunrise to noon the process is similar but reversed, from reddish to yellowish. Thus, depending on the time of day when sunlight illuminates a road, the red component of the light reflected from the road surface can be considered to be affected by a similar or a higher quantity of light than the green, whereas the blue intensity of sunlight is the lowest, i.e.,

(6) $$\begin{array}{c} E_{sun}\left(\lambda_{R}\right)\geq E_{sun}\left(\lambda_{G}\right),\\ E_{sun}\left(\lambda_{R}\right)>E_{sun}\left(\lambda_{B}\right),\;\\ E_{sun}\left(\lambda_{G}\right)>E_{sun}\left(\lambda_{B}\right), \end{array}$$

Based on the reflection model and both skylight and sunlight contributions to the surface chromaticity, we propose the above set of three chrominance properties of shadows.

## 3. New Shadow Features

According to the reflection model, when comparing a pixel in the shadowed region (*x_sha_*, *y_sha_*) to a non-shadowed one (*x_non-sha_*, *y_non-sha_*) of the same material surface in an image, the contribution of sunlight on (*x_non-sha_*, *y_non-sha_*) according to the reflection model is given by

(7) $$C_{i}{\left(x_{non-sha},y_{non-sha}\right)}_{sun}=C_{i}(x_{non-sha},y_{non-sha})-C_{i}(x_{sha},y_{sha}),$$

giving a color vector *C_i_*(*x_non-sha_*, *y_non-sha_*)*_sun_* = [*R_sun_*, *G_sun_*, *B_sun_*].

**Property 1.** Considering the red and green components of skylight and sunlight, a relationship between a shadowed region and a non-shadowed one of the same material surface is proposed by taking into account the different components dominating the illumination. From Equation (4), the red and green components of a pixel in a shadowed region are respectively

(8) $$R_{sha}=E_{sky}\left(\lambda_{R}\right)\times S\left(\lambda_{R}\right)\times q_{R},\;G_{sha}=E_{sky}\left(\lambda_{G}\right)\times S\left(\lambda_{G}\right)\times q_{G},$$

By taking a ratio of the two components, the red and green surface reflectances are related by

(9) $$\frac{R_{sha}}{G_{sha}}=\frac{E_{sky}\left(\lambda_{R}\right)\times S\left(\lambda_{R}\right)\times q_{R}}{E_{sky}\left(\lambda_{G}\right)\times S\left(\lambda_{G}\right)\times q_{G},}\Rightarrow \frac{S\left(\lambda_{R}\right)\times q_{R}}{S\left(\lambda_{G}\right)\times q_{G}}=\frac{E_{sky}\left(\lambda_{G}\right)}{E_{sky}\left(\lambda_{R}\right)}\times \frac{R_{sha}}{G_{sha}}.$$

Similarly, the contribution of sunlight on the red and green components of the non-shadowed surface is obtained from Equation (3) by making *E_sky_*(*λ_i_, x, y*) = 0, i.e.,

(10) $$R_{sun}=E_{sun}\left(\lambda_{R}\right)\times S\left(\lambda_{R}\right)\times q_{R},\;G_{sun}=E_{sun}\left(\lambda_{G}\right)\times S\left(\lambda_{G}\right)\times q_{G}.$$

Taking a ratio of the two components gives

(11) $$\frac{R_{sun}}{G_{sun}}=\frac{E_{sun}\left(\lambda_{R}\right)\times S\left(\lambda_{R}\right)\times q_{R}}{E_{sun}\left(\lambda_{G}\right)\times S\left(\lambda_{G}\right)\times q_{G}}\Rightarrow \frac{S\left(\lambda_{R}\right)\times q_{R}}{S\left(\lambda_{G}\right)\times q_{G}}=\frac{E_{sun}\left(\lambda_{G}\right)}{E_{sun}\left(\lambda_{R}\right)}\times \frac{R_{sun}}{G_{sun}}.$$

Equating Equations (9) and (11) yields

(12) $$\frac{E_{sky}\left(\lambda_{G}\right)}{E_{sky}\left(\lambda_{R}\right)}\times \frac{R_{sha}}{G_{sha}}=\frac{E_{sun}\left(\lambda_{G}\right)}{E_{sun}\left(\lambda_{R}\right)}\times \frac{R_{sun}}{G_{sun}}\Rightarrow \frac{E_{sky}\left(\lambda_{G}\right)}{E_{sky}\left(\lambda_{R}\right)}\times \frac{E_{sun}\left(\lambda_{R}\right)}{E_{sun}\left(\lambda_{G}\right)}=\frac{G_{sha}}{R_{sha}}\times \frac{R_{sun}}{G_{sun}}.$$

According to Equation (5), the green component of the skylight is generally equal to or slightly higher than the red one, whereas, according to Equation (6), the red component of the sunlight is generally equal to or higher than the green; thus, the left-hand side of Equation (12) satisfies

(13) $$\;\frac{E_{sky}\left(\lambda_{G}\right)}{E_{sky}\left(\lambda_{R}\right)}\times \frac{E_{sun}\left(\lambda_{R}\right)}{E_{sun}\left(\lambda_{G}\right)}\geq 1.$$

According to Equations (12) and (13), when comparing a pixel in the shadow to a non-shadowed pixel of the same material surface, the following constraint is satisfied:

(14) $$\frac{G_{sha}}{R_{sha}}\times \frac{R_{sun}}{G_{sun}}\geq 1,$$

where *R_sun_* = *R*(*x_non-sha_*, *y_non-sha_*) − *R*(*x_sha_*, *y_sha_*) and *G_sun_* = *G*(*x_non-sha_*, *y_non-sha_*) − *G*(*x_sha_*, *y_sha_*). This equation shows that the relationship between the red and green surface reflectances due to sunlight is equal to or higher than the red–green one due to skylight.

**Figure 2:**
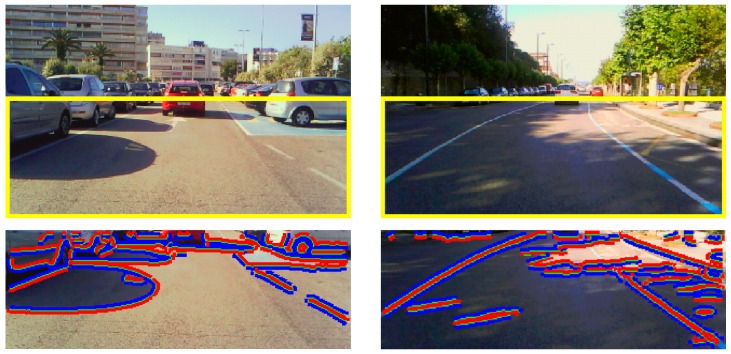
(**First row**) Bounding box containing the region of interest (ROI) of two input red–green–blue (RGB) images of our dataset. (**Second row**) Darker (in blue) and brighter (in red) regions on both sides of each edge (in green) of the ROI image.

**Figure 3:**
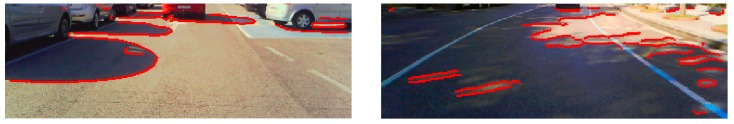
Shadow edge detection of images in Figure 2 obtained using Property 1, i.e., Equation (14).

The first row of Figure 2 illustrates two representative traffic scenes of our dataset, where a region of interest (ROI) focusing on the road is overlaid onto the images. The second row of Figure 2 shows the edges (in green) of the ROI image, as well as the two regions across them. The darker region (in blue) of each edge is candidate to be a shadow, whereas the brighter one (in red) is assumed the non-shadowed reference region. The method to determine the edges, the darker and brighter regions, and the surface reflectance values of the regions is described in detail in Section 4. Figure 3 illustrates the detected shadow obtained using the constraint of Property 1, i.e., Equation (14). Those edges where the surface reflectances corresponding to the dark (*C_i_*(*x_sha_*, *y_sha_*) = [*R_sha_*, *G_sha_*, *B_sha_*]) and bright (*C_i_*(*x_non-sha_*, *y_non-sha_*) = [*R_non-sha_*, *G_non-sha_*, *B_non-sha_*) regions satisfy Equation (14) are classified as shadow edges (in red). Otherwise, the edges are classified as edges due to a material change and are removed.

**Property 2.** Three constraints are introduced to consider the effect of sunlight on neutral surfaces. Asphalt roads are generally neutral surfaces, which present similar reflectance for each component (*S*(*λ_R_*) ≈ *S*(*λ_B_*) ≈ *S*(*λ_G_*)); thus, their RGB distribution is practically proportional to the SPD of the incident illumination. This implies that the reflectance components of a non-shadowed neutral surface due to the sunlight contribution are proportional to the red, green, and blue components of sunlight. The RGB reflectance components of a non-shadowed pixel due to sunlight are obtained from Equation (3) by making *E_sky_*(*λ_i_, x, y*) = 0. Taking the ratios of two components gives

(15) $$\frac{R_{sun}}{G_{sun}}=\frac{E_{sun}\left(\lambda_{R}\right)\times S\left(\lambda_{R}\right)\times q_{R}}{E_{sun}\left(\lambda_{G}\right)\times S\left(\lambda_{G}\right)\times q_{G}},\;\frac{R_{sun}}{B_{sun}}=\frac{E_{sun}\left(\lambda_{R}\right)\times S\left(\lambda_{R}\right)\times q_{R}}{E_{sun}\left(\lambda_{B}\right)\times S\left(\lambda_{B}\right)\times q_{B}},\;\frac{G_{sun}}{B_{sun}}=\frac{E_{sun}\left(\lambda_{G}\right)\times S\left(\lambda_{G}\right)\times q_{G}}{E_{sun}\left(\lambda_{B}\right)\times S\left(\lambda_{B}\right)\times q_{B}},$$

Assuming *S*(*λ_R_*) = *S*(*λ_B_*) = *S*(*λ_G_*) and considering, in line with References [6,12,41,47], similar spectral sensitivity constants of the three color camera sensors (i.e., *q_R_* = *q_G_* = *q_B_*), Equation (15) becomes

(16) $$\frac{R_{sun}}{G_{sun}}=\frac{E_{sun}\left(\lambda_{R}\right)}{E_{sun}\left(\lambda_{G}\right)},\;\frac{R_{sun}}{B_{sun}}=\frac{E_{sun}\left(\lambda_{R}\right)}{E_{sun}\left(\lambda_{B}\right)},\;\frac{G_{sun}}{B_{sun}}=\frac{E_{sun}\left(\lambda_{G}\right)}{E_{sun}\left(\lambda_{B}\right)}$$

Thus, taking into account the SPD of the yellow sunlight which presents a red component higher or equal to the green (depending on the time of day) and higher than the blue, as well as a green component higher than the blue (i.e., Equation (6)), the RGB reflectances of the non-shadowed surface due to sunlight contribution satisfy the three constraints

(17) $$\frac{R_{sun}}{G_{sun}}\geq 1,\;\frac{R_{sun}}{B_{sun}}>1,\;\frac{G_{sun}}{B_{sun}}>1\;.$$

**Figure 4:**
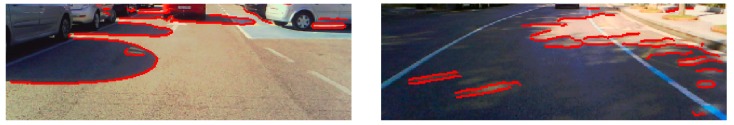
Shadow edge detection obtained by the three constraints in Property 2.

The effect of illumination on neutral surfaces was first considered in Reference [39], where the focus was on the skylight contribution to the shadowed road regions. In that case, the blue component of shadows was dominant over the red and green ones; thus, the constraints *R_sha_* > *G_sha_* and *R_sha_* > *B_sha_* were satisfied in the umbra of shadows, which was illuminated only by skylight. However, the dominance of the blue light in penumbras was not so strong because they were also illuminated by some amount of sunlight. Thus, even low-saturated surfaces such as asphalt road surface may maintain their dominant color component when softly shadowed. However, the constraints in Equation (17) are also satisfied when comparing penumbras to non-shadowed road, since they do not focus on the skylight contribution to the shadowed road surface but on the sunlight contribution to the non-shadowed one. Figure 4 illustrates the shadow detection of the images in Figure 2 after applying the three constraints associated with Property 2, i.e., Equation (17).

**Property 3.** A set of two relationships is introduced to take into account both the similarity of the red and green components of the illumination and the large variation of the blue component. For both skylight and sunlight, the red and green intensities are close to each other. Thus, the relationship between the red and green components reflected off the surface illuminated by skylight is close to that due to sunlight. However, the red and blue intensities are very different in magnitude and sign (i.e., *E_sky_*(*λ_R_*) < *E_sky_*(*λ_B_*), *E_sun_(λ_R_)* ≥ *E_sun_*(*λ_G_*)). Thus, the relationship red–blue of the surface illuminated by skylight is significantly different from that due to sunlight.

Focusing on neutral surfaces and assuming *S*(*λ_R_*) = *S*(*λ_B_*) = *S*(*λ_G_*), as well as *q_R_* = *q_G_* = *q_B_* as in Property 2, the red component proportion of the surface related to the green, i.e., *rg,* and to the blue, i.e., *rb*, can be expressed as

(18) $$rg=\frac{R}{R+G}=\frac{E\left(\lambda_{R}\right)\times S\left(\lambda_{R}\right)\times q_{R}}{E\left(\lambda_{R}\right)\times S\left(\lambda_{R}\right)\times q_{R}+E\left(\lambda_{G}\right)\times S\left(\lambda_{G}\right)\times q_{G}}=\frac{E\left(\lambda_{R}\right)}{E\left(\lambda_{R}\right)+E\left(\lambda_{G}\right)},\;rb=\frac{R}{R+B}=\frac{E\left(\lambda_{R}\right)\times S\left(\lambda_{R}\right)\times q_{R}}{E\left(\lambda_{R}\right)\times S\left(\lambda_{R}\right)\times q_{R}+E\left(\lambda_{B}\right)\times S\left(\lambda_{B}\right)\times q_{B}}=\frac{E\left(\lambda_{R}\right)}{E\left(\lambda_{R}\right)+E\left(\lambda_{B}\right)}.$$

When comparing a shadowed region to a non-shadowed one of the same material surface, the difference in the red–green and red–blue proportions due to skylight and sunlight are respectively

(19) $$\left|rg_{sha}-rg_{sun}\right|=\left|\frac{R_{sky}}{R_{sky}+G_{sky}}-\frac{R_{sun}}{R_{sun}+G_{sun}}\right|=\left|\frac{E_{sky}\left(\lambda_{R}\right)}{E_{sky}\left(\lambda_{R}\right)+E_{sky}\left(\lambda_{G}\right)}-\frac{E_{sun}\left(\lambda_{R}\right)}{E_{sun}\left(\lambda_{R}\right)+E_{sun}\left(\lambda_{G}\right)}\right|,\;\left|rb_{sha}-rb_{sun}\right|=\left|\frac{R_{sky}}{R_{sky}+B_{sky}}-\frac{R_{sun}}{R_{sun}+B_{sun}}\right|=\left|\frac{E_{sky}\left(\lambda_{R}\right)}{E_{sky}\left(\lambda_{R}\right)+E_{sky}\left(\lambda_{B}\right)}-\frac{E_{sun}\left(\lambda_{R}\right)}{E_{sun}\left(\lambda_{R}\right)+E_{sun}\left(\lambda_{B}\right)}\right|.$$

According to Equation (5), the green component of the skylight is smaller than the blue one (i.e., *E_sky_*(*λ_G_*) < *E_sky_*(*λ_B_*)), whereas, from Equation (6), the green component of the sunlight is higher than the blue (i.e., *E_sun_*(*λ_G_*) > *E_sun_*(*λ_B_*)); thus,

(20) $$\frac{E_{sky}\left(\lambda_{R}\right)}{E_{sky}\left(\lambda_{R}\right)+E_{sky}\left(\lambda_{G}\right)}>\frac{E_{sky}\left(\lambda_{R}\right)}{E_{sky}\left(\lambda_{R}\right)+E_{sky}\left(\lambda_{B}\right)}\Rightarrow rg_{sha}>rb_{sha},\;\frac{E_{sun}\left(\lambda_{R}\right)}{E_{sun}\left(\lambda_{R}\right)+E_{sun}\left(\lambda_{B}\right)}<\frac{E_{sun}\left(\lambda_{R}\right)}{E_{sun}\left(\lambda_{R}\right)+E_{sun}\left(\lambda_{B}\right)}\Rightarrow rg_{sun}<rb_{sun}.$$

This implies that the change in the red–green proportion due to skylight and sunlight is smaller than the change in the red–blue one, i.e., the following first constraint of Property 3:

(21) $$\left|rg_{sha}-rg_{sun}\right|<\left|rb_{sha}-rb_{sun}\right|\Rightarrow \frac{\left|rg_{sha}-rg_{sun}\right|}{\left|rb_{sha}-rb_{sun}\right|}<1.$$

This reasoning is also valid when comparing the changes of the surface relationships green–red and green–blue. From Equation (5), the red component of the skylight is smaller than the blue (i.e., *E_sky_*(*λ_R_*) < *E_sky_*(*λ_B_*)), whereas, from Equation (6), the red component of the sunlight is higher than the blue (i.e., *E_sun_*(*λ_R_*) > *E_sun_*(*λ_B_*)); thus, *gr_sha_* > *gb_sha_* and *gr_sun_* < *gb_sun_*. Therefore, the change in the green–red proportion due to skylight and sunlight is smaller than the change in the green–blue one, i.e., the following second constraint of Property 3:

(22) $$\left|gr_{sha}-gr_{sun}\right|<\left|gb_{sha}-gb_{sun}\right|\Rightarrow \frac{\left|gr_{sha}-gr_{sun}\right|}{\left|gb_{sha}-gb_{sun}\right|}<1.$$

**Figure 5:**
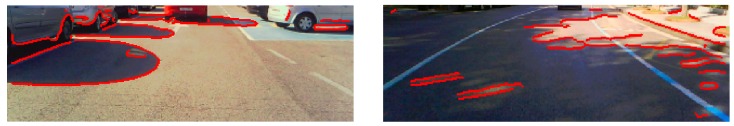
Shadow edge detection obtained by the constraint which relates the red–green and the red–blue proportions of Property 3, i.e., Equation (21).

**Figure 6:**
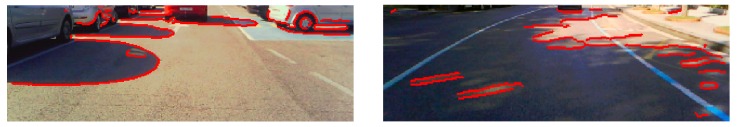
Shadow edge detection obtained by the constraint which relates the green–red and the green–blue proportions of Property 3, i.e., Equation (22).

For simplicity, Equations (21) and (22) are obtained considering neutral surface conditions (i.e., *S*(*λ_R_*) = *S*(*λ_B_*) = *S*(*λ_G_*)); thus, they are especially applicable to asphalt roads which are generally colorless surfaces with similar reflectance for each component. Figure 5 illustrates the shadow edge detection for both scenes in Figure 2 using the constraint associated with Property 3, which relates the red–green and red–blue proportions of the surface, i.e., Equation (21). Figure 6 shows, on the other hand, the shadow edge detection using the constraint associated with Property 3 relating the green–red and green–blue proportions, i.e., Equation (22).

**Figure 7:**
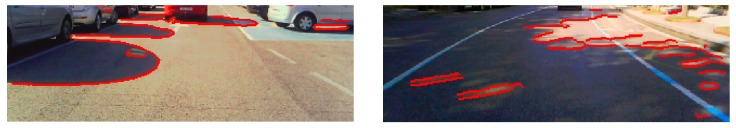
Shadow edge detection results obtained after applying the three properties all together.

Figure 7 illustrates the shadow edge detection after applying the proposed three properties, i.e., Equations (14), (17), (21) and (22). As can be observed, the accumulation of shadow feature constraints contributes to a better characterization of shadows since the possibility of errors in the classification of edges due to a material change decreases.

## 4. Shadow Edge Detection Method

On the basis of the new chrominance properties of shadows, a shadow detection method for onboard road detection is proposed. As long as onboard systems deal with still images, there is not a known non-shadowed reference road region in the incoming image to compare the pixel properties. Thus, the shadow detection method focuses on comparing pixel properties across image edges, where the darker region of an image edge is the candidate shadow region and the brighter one is assumed to be the non-shadowed reference region. The method comprises four main stages: extraction of the image edges, selection of the bright and dark regions across the edges, extraction of the strong edges, and shadow edge classification.

In the image edge extraction stage, we break T- and X-junctions that connect different edges, thus obtaining an edge map consisting of individual edges. In order to achieve robustness in the edge classification, we exploit regions across each edge instead of single pixels. Thus, in line with Reference [22], we use two regions of pixels along both sides of the edges to compute the chrominance properties of the surface. Prior to edge classification, an intensity filtering is applied to eliminate noisy edges on the asphalt, thus retaining only the strong ones in the image. Finally, edge classification is carried out by verifying whether the regions on both sides of the strong edges satisfy the six constraints associated with the proposed three chrominance properties of shadow, thus classifying each image edge as a shadow edge or a material-change edge.

**Figure 8:**
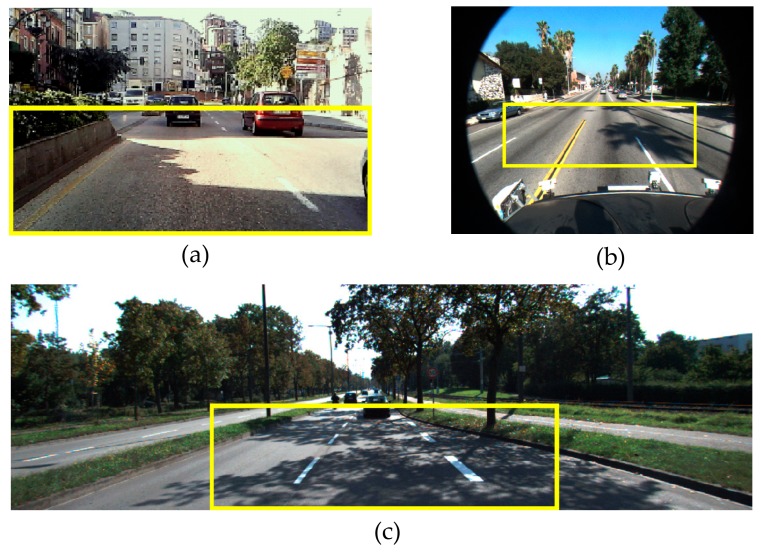
Bounding box containing the ROI in the incoming color images. (**a**) For our 240 × 320 camera, the ROI covers 110 × 320 pixels. (**b**) For the Caltech Lane dataset containing 480 × 640 images, the ROI considered covers 130 × 410 pixels. (**c**) For the Kitti dataset containing 375 × 1242 images, the ROI considered covers 170 × 574 pixels.

Since the method addresses the detection of shadow edges on the road, in order to simplify a captured road scene, as well as reduce the number of false positive detections outside the road surface, an ROI in the incoming color images is defined on the road by using knowledge of the scene perspective and assuming flat road surface as in References [4,59]. The camera is installed beside the rear-view mirror of the ego-vehicle, and the ROI is a rectangular area covering the road region ahead, excluding most of the image areas which do not correspond with the ground (see Figure 8).

### 4.1. Extraction of the Image Edges

**Figure 9:**
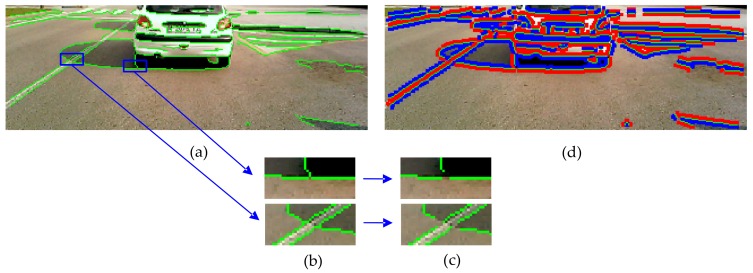
(**a**) Canny edge map (in green) overlaid on the ROI of an incoming image of our dataset. (**b**) Two examples of T-junction connecting different edges. (**c**) Individual edges after removing T-junctions. (**d**) Brighter (in red) and darker (in blue) regions across edges (in green) after T- and X-junction removal.

**Figure 10:**
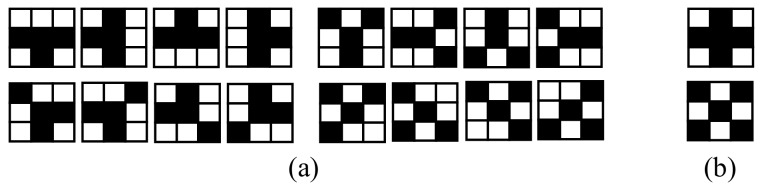
Masks used for the detection of (**a**) T-junction and (**b**) X- junction.

After an averaging low-pass filtering to reduce image noise, the edges in the ROI of the incoming RGB image are extracted by applying the Canny operator [60] owing to its robustness. The resulting edge map consists of edges due to both shadow boundaries and material changes (see Figure 9a). However, a troublesome effect of the edge extraction is the generation of T- and X-junctions, which affect the shadow edge classification since they connect different edges (see Figure 9b). The edge classification requires separating edges into two regions only; thus, individual edges are generated by removing X- and T-junctions. To this end, the edge map is scanned bottom-up and a 3 × 3 kernel centered on each edge pixel is matched with a total of 18 T- and X-masks, as shown in Figure 10. For a positive match, a junction is broken by removing from the edge map the pixels involved in the junction (see Figure 9c). The result is an edge map consisting of individual edges that only separate two regions of the image (see Figure 9d, where the edges are in green).

### 4.2. Extraction of the Bright and Dark Regions across the Edges

Since pixel-based methods that use information of a pixel or a small neighborhood around a pixel are prone to noise [44], we impose spatial consistency which employs a higher-level neighborhood on both sides of each edge. To this end, we compute the gradient orientation of each pixel of the edge and consider three pixels along this orientation and on both sides. This operation is performed for all the pixels of the edge, thus obtaining two different regions across each edge. The reflectance components of the darker (*R_sha_*, *G_sha_*, *B_sha_*) and brighter (*R_non-sha_*, *G_non-sha_*, *B_non-sha_*) regions are computed as the mean pixel values of each region as in Reference [22]. The region with the larger mean pixel values (the brighter) is assumed the non-shadowed reference region of the edge, whereas the region with the smaller values (the darker) is assumed the candidate shadow region. Figure 9d illustrates both regions across each edge of the image, where the blue areas represent the darker regions and the red areas represent the brighter ones. If a pixel of a region is also an edge pixel of a different boundary of the image, it is not included in the mean value computation. This may happen with edges three or fewer pixels away.

### 4.3. Extraction of Strong Edges

**Figure 11:**
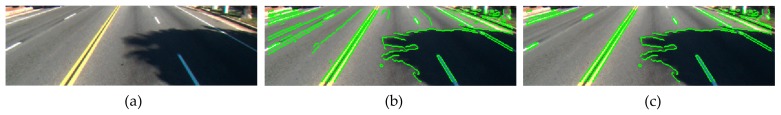
(**a**) ROI of an input image of the Caltech Lane dataset. (**b**) ROI overlaid with Canny edge map. (**c**) Enhanced edge map *D* after applying the intensity filter, i.e., Equation (23).

Asphalt roads are generally textured surfaces that usually generate noisy edges in the image. The regions on both sides of a noisy edge fall onto the road; thus, the intensity difference between them is generally small. Moreover, depending on the illumination and type of asphalt, the intensity difference between a shadowed road region and a non-shadowed one vary. However, their difference is generally significant. In order to discard a noisy edge on the road, a filtering strategy based on the intensity difference between both sides of the edge is proposed. An image edge is removed from the edge map if the intensity difference between the regions on both sides of the edge *I_sun_* is smaller than the 20% of the intensity of the darker region *I_sha_,* i.e.,

(23) $$I_{sun\;}<\;0.2\;\times \;I_{sha},$$

where *I_sun_* = *I_non-sha_* − *I_sha_*, *I_non-sha_* = (*R_non-sha_* + *G_non-sha_* + *B_non-sha_*)/3 and *I_sha_* = (*R_sha_* +*G_sha_* +*B_sha_*)/3. The choice of 20% of the shadowed region intensity is conservative because the aim of the filter is not to identify shadow edges but discard those whose small intensity difference does not clearly correspond to the intensity difference across a shadow edge (see Figure 11).

### 4.4. Shadow Edge Classification

Edge classification is the final stage of the method, where each individual edge *D_k_* is classified as a shadow edge if the reflectance components of its darker (*R_sha_*, *G_sha_*, *B_sha_*) and brighter (*R_non-sha_*, *G_non-sha_*, *B_non-sha_*) regions satisfy the six constraints associated with the three chrominance properties of shadow, i.e., the six chrominance constraints in Equations (14), (17), (21) and (22). Otherwise, if one of the constraints is not satisfied, then the edge is classified as an edge due to a material change, i.e.,

(24) $$D_{k}=\left|\begin{array}{ccc} \mathrm{if} & property\;1 & \\ & \mathrm{and}\;property\;2 & \\ & \mathrm{and}\;property\;3, & \mathrm{Shadow}\;\mathrm{Edge},\\ \mathrm{otherwise}, & & \mathrm{Material}\;\mathrm{Change}\;\mathrm{Edge}. \end{array}\right.$$

## 5. Experimental Results

We firstly discuss the individual performance of each of the proposed three chrominance properties and then the complete shadow edge detection method. In addition, we compare the proposed method with four state-of-the-art shadow detection methods. In our experiments, we used image sequences acquired using an onboard camera which provided 240 × 320 color image frames with an 8-bit pixel depth. A total of 6600 road images in 22 sets of 300 frames were acquired in real traffic. The data consist of urban traffic scenes in the presence of a variety of cast shadows on the road and scenes which do not contain shadows. We also used the publicly available Caltech Lane dataset [61] for driving assistance systems, which consists of 1225 PNG road images of 480 × 640, and the Kitti Road dataset [62] for road and lane detection, which includes 579 PNG images of 175 × 1242 pixels captured under different illumination.

### 5.1. Individual Performance of the Proposed Shadow Properties

#### 5.1.1. Qualitative Results

**Figure 12:**
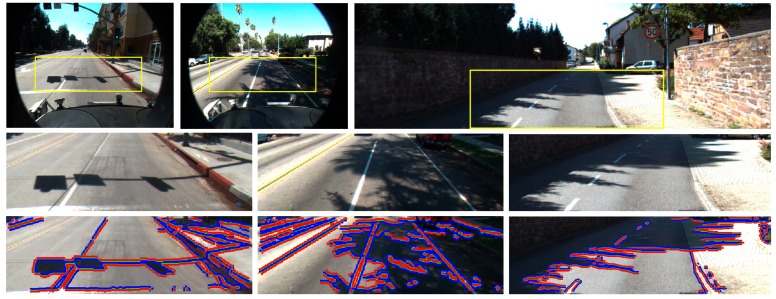
(**Row 1**) input images; (**Row 2**) ROI of the input images; (**Row 3**) ROI overlaid with brighter (red) and darker (blue) regions across strong edges (green).

Figure 12 illustrates two representative traffic scenes of the Caltech Lane dataset (left and middle images) and one scene of the Kitti Road dataset (right image), which cover a selection of different types of shadows. The image on the left contains a very dark shadow caused by the traffic light which generates well-defined shadow edges on the road. The image in the middle contains shadows caused by the branches of the palm tree which generate soft shadow boundaries. In the image on the right, the trees cause shadows with both well-defined and soft edges on the road.

**Figure 13:**
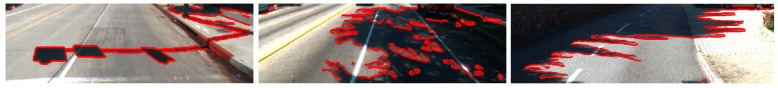
Shadow edge detection results of images in Figure 12 obtained by Property 1.

**Property 1.**Figure 13 shows the shadow edge detection results using the constraint associated with Property 1, i.e., Equation (14). As can be observed in the three images, both the well-defined and the soft shadow boundaries are successfully identified as shadow edges, whereas most of the edges caused by material changes such as those caused by lane markings and curbs are also correctly classified and removed from shadow edge map. In the three images, the number of misclassified edges due to material changes is small, and they occur in image regions outside the road. In the right side of the middle image, the material-change edge caused by the grass and the sidewalk is misclassified as shadow edge and thus retained in the shadow edge map. Note that the Canny edge detector provides one-pixel-thick edges; however, the thickness of the edges in the images was increased for a better visualization.

**Figure 14:**
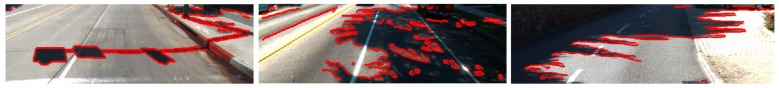
Shadow edge detection results of images in Figure 12 obtained by Property 2.

**Property 2.**Figure 14 shows the shadow edge detection results using the three constraints associated with Property 2, i.e., Equation (17). As can be observed in the three images, Property 2 also demonstrates effectiveness in the detection of both well-defined and soft shadow edges. However, despite of the fact that the number of misclassified boundaries due to material changes is also small, there are some errors in the classification of strong noise edges on the asphalt road that remained after the intensity filtering. In the middle image, two noisy edges on the asphalt are falsely classified as shadow edges. In fact, they are not material change edges since they do not separate two different materials but two regions with different reflectance of a same surface. On the other hand, in the middle image, it can be observed that the material-change edge caused by the grass and the sidewalk is successfully identified as a material-change edge.

**Figure 15:**
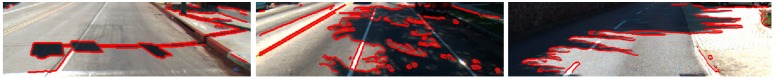
Shadow edge detection results of images in Figure 12 obtained by Property 3.

**Property 3.**Figure 15 shows the shadow edge detection results using both constraints associated with Property 3, i.e., Equations (21) and (22). The ability to detect both well-defined and soft shadow edges is clearly shown. However, the effectiveness in classifying material-change edges decreases when compared with using Properties 1 and 2. Some edges on the road region due to lane markings and curbs, as well as material-change edges outside the road, are misclassified as shadow edges.

#### 5.1.2. Quantitative Results

**Figure 16:**
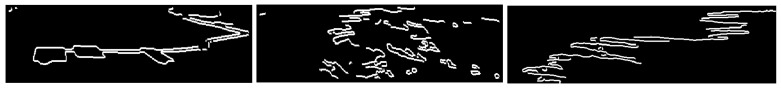
Ground-truth shadow edge maps of images in Figure 12.

In order to quantitatively evaluate the performance of the proposed three chrominance properties, we compute the commonly used metrics of precision, recall, and F-measure, i.e.,

(25) $$\mathrm{Precision}=\frac{TP}{TP+FN},\;\;\mathrm{Recall}=\frac{TP}{TP+FP},\;\;F-\mathrm{measure}=2\times \frac{\mathrm{Precision}\times \mathrm{Recall}}{\mathrm{Precision}+\mathrm{Recall}},$$

where *TP* (true positive) is the number of pixels correctly detected as shadow edges, *FP* (false positive) is the number of pixels due to material changes misclassified as shadow edges, and *FN* (false negative) is the number of pixels due to shadow edges misclassified as material-change edges. Higher values of precision, recall, and F-measure denote better results. The evaluation was performed on 300 images consisting of 100 images of the Caltech Lane dataset, 100 images of the Kitti Road dataset, and 100 images of our dataset. The set of images for evaluation includes road scenes captured under different illumination in the presence of a variety of cast shadows on the road, as well as scenes which do not contain shadows. *TP*, *FP*, and *FN* are determined by a pixel-wise comparison between the resulting shadow edge map obtained using the property under evaluation and the ground-truth shadow edge map manually extracted (see Figure 16).

Table 1 shows the metrics of each of the properties evaluated on each of the 300 images. The table shows that the precision of the three properties on each dataset is high, achieving values of 0.950, 0.959, and 0.932, respectively. This indicates high effectiveness of each property in the classification of shadow edges, which in turn implies a high number of true positives together with a low number of false negatives.

Table 1 shows for each property that the recall values are lower than the precision ones, achieving 0.730, 0.737, and 0.706, respectively. The recall is an indicator of the effectiveness in the classification of material-change edges. A higher recall suggests fewer misclassified material-change edges (false positives). Thus, lower recall values indicate that, in addition to shadow edges, the proposed properties can also be satisfied by some material changes. The recall values decrease the F-measure, achieving values of 0.826, 0.833, and 0.804, respectively.

Table 1 also shows that Property 2 achieves the highest precision (0.959), recall (0.737), and F-measure (0.833), which indicates that it is the most robust property. The precision due to Property 3 is also high (0.932) but the recall (0.706) is the lowest, which makes Property 3 effective in shadow edge detection but least reliable in the classification of edges due to a material change.

Table 1 shows that the three chrominance properties achieve better results on the Caltech Lane and Kitti Road datasets, but the lowest values are obtained on ours. This is because the Caltech Lane and Kitti Road datasets comprise better-quality images with higher definition.

From the qualitative and quantitative results, we can extract three main conclusions:

1. The three chrominance properties demonstrate their effectiveness in identifying shadow edges, validating the considerations made in the reflectance model, the SPD of the illumination, and the properties.

2. Each property demonstrates its effectiveness in the detection of both well-defined and soft shadow edges (penumbras). This is because each property focuses on the sunlight contribution to the non-shadowed road, which occurs whether the latter is compared with the umbra or penumbra road regions (external shadow contours) or when the umbra is compared with the penumbra (internal shadow contours in the middle image of Figure 13, Figure 14 and Figure 15).

3. The three chrominance properties can be satisfied by some edges separating material changes, i.e., false positives; thus, the individual application of each of them is not sufficient to unequivocally classify an image edge.

### 5.2. Performance of the Proposed Shadow Edge Detection Method

#### 5.2.1. Qualitative Results

**Figure 17:**
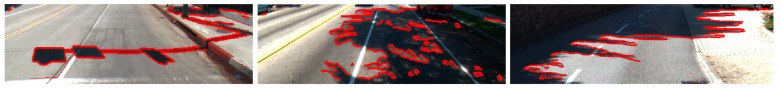
Shadow edge detection results of images in Figure 12 obtained by the proposed method.

Figure 17 shows the shadow edge detection results of the images in Figure 12 after applying the proposed method, which incorporates the six constraints associated with Properties 1, 2, and 3, i.e., Equation (24). The results show that both the well-defined and the soft shadow edges are successfully identified as shadow edges. The majority of boundaries caused by material changes are also correctly classified and removed from shadow edge maps.

In the proposed method, an edge has to satisfy the six constraints to be classified as shadow edge; otherwise, the edge is classified as material change. On the one hand, the accumulation of properties makes the effectiveness of the method in the shadow edge classification decrease in relation to the effectiveness of each property. Each individual property provides shadow edges (i.e., true positives); however, they do not have to be the same for the three properties. Thus, the number of shadow edges correctly classified by the method is lower than that provided by each individual property. However, as the three properties demonstrate high effectiveness in the shadow edge detection, the effectiveness of the method is consequently high. In addition, the fact that a shadow edge has to satisfy the six constraints makes the shadow edge detection very reliable.

**Figure 18:**
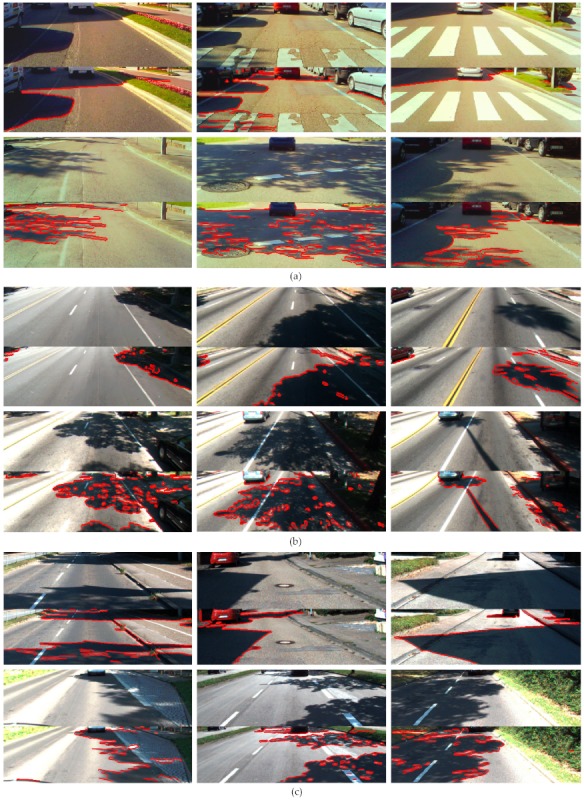
Example results of the proposed shadow edge detection method on (**a**) images of our dataset, (**b**) images of the Caltech Lane dataset, and (**c**) images of the Kitti Road dataset.

On the other hand, the accumulation of constraints makes the material-change edge classification more effective than each individual property. An edge is classified as a material-change edge if just one of the six constraints associated with the three properties is not satisfied. Each individual property provides false detections; however, the false detections do not have to be the same for each property (e.g., in the middle image of Figure 12, the material-change edge caused by the grass and the pavement is misclassified as a shadow edge by Properties 1 and 3; however, it is correctly classified as material change by Property 2). Thus, if just one constraint correctly classifies a material change, the method accepts it regardless of the results due to the other five constraints. Figure 17 shows the reduction of misclassified material-change edges when compared to Figure 13, Figure 14 and Figure 15. To better show the performance of the method, Figure 18 shows some example results in challenging road scenes of the three datasets.

#### 5.2.2. Quantitative Results

The bottom row of Table 1 shows the results of the complete shadow edge detection method on each of the three datasets. It shows that the precision, recall, and F-measure on each dataset are high, achieving values of 0.905, 0.884, and 0.870, respectively, on the 300 images. Furthermore, the precision value achieved by the method (0.905) is lower than that of each individual property, whereas the recall value (0.884) is higher. The F-measure value achieved by the method (0.870) indicates effectiveness not only in the classification of shadow edge classification but also material change.

From the qualitative and quantitative results of the proposed method, we can extract two main conclusions:

1. The proposed shadow edge detection method demonstrates effectiveness in identifying shadow edges, achieving a precision value of 0.905. In addition, the accumulation of chrominance properties makes the shadow edge detection more reliable since a shadow edge has to satisfy the six constraints.

2. The accumulation of shadow properties improves the effectiveness in the classification of material-change edges, making the method achieve recall and F-measure values of 0.884 and 0.870, respectively.

#### 5.2.3. Results on Images without Shadows

**Figure 19:**
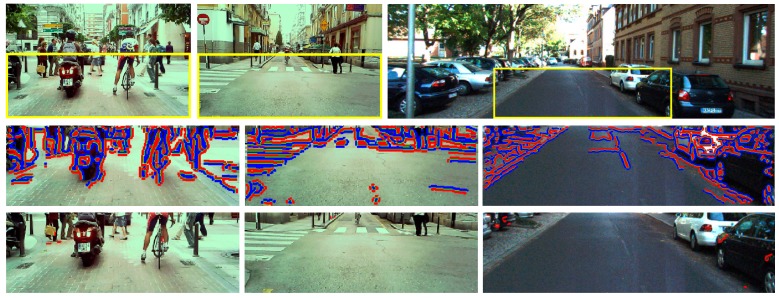
(**Row 1**) input images; (**Row 2**) ROI overlaid with brighter (red) and darker (blue) regions across strong edges (green); (**Row 3**) results of the proposed shadow edge detection method.

An effective shadow detection method does not only detect shadows but also does not provide false detections in scenes that do not contain shadows. Figure 19 illustrates two cluttered traffic scenes of our dataset (left and middle images) and one scene of the Kitti Road dataset (right image), which cover two different types of illumination that do not cause shadows on the road. The images on the left and in the middle were captured under cloudy conditions, whereas, in the image on the right, the ego-vehicle is traveling along a street in the shade. As can be observed in the three images, most of the edges caused by material changes are correctly classified and removed from shadow edge map. The number of false detections is small, and they occur in image regions outside the road surface. In the left image, the material-change edges caused by the pedestrians, cyclists, a motorcyclist, and manhole covers on the road are successfully classified as material changes. In the middle image, the material-change edges due to the pedestrian crossing, pedestrians, and curbs are also correctly identified. In the right image, most of the material-change edges caused by the road boundaries, asphalt noise, and vehicles are correctly detected, except for some noisy edges caused by some reflection on vehicles. However, it can be observed that the shadows underneath both vehicles on the right are misclassified as material change and removed from the image. Overall, Figure 19 shows the effectiveness of the proposed method in identifying of material-change edges in shadow-free images.

#### 5.2.4. Limitations

The experiments demonstrate effectiveness of the proposed method in the classification of both shadow edges and material changes. However, there are three limitations.

**Figure 20:**
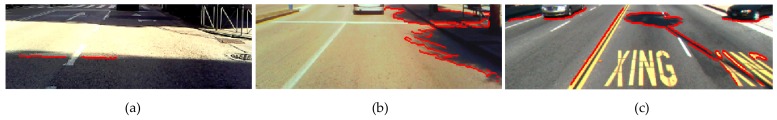
Detection errors: (**a**) oversaturated road region; (**b**) shadow underneath a vehicle; (**c**) yellow marking edges.

**Limitation 1.** Overexposed image regions may lead to shadow edge misclassification. Sunlight may cause oversaturated road regions in the image, whose RGB components saturate to a gray level of 255; thus, the surface chrominance is undermined. Figure 20a shows the over-exposure problem where the RGB components of the non-shadowed road are saturated leading to false negatives, i.e., misclassification of shadow edges. This problem is inherent to any physics-based method and could be mitigated using cameras of higher dynamic range.

**Limitation 2.** Owing to the lack of light underneath a vehicle, the shadow underneath is generally a very dark road region whose RGB reflectance components have very low values. Unlike over-exposed road regions, the shadow underneath a vehicle may be saturated to the minimum value of the range, i.e., 0, making the surface chrominance feature unstable [59]. Some examples of Figure 18 and Figure 19c, and the middle image of Figure 20 show this problem, resulting in misclassification of edges due to the shadow underneath. However, for some applications such as road detection and front vehicle detection, this misclassification is in fact a positive outcome as the edge of the shadow underneath a vehicle is the bottom part of its contour [59].

**Limitation 3.** In some cases, edges due to yellow markings on the road may satisfy the proposed shadow constraints, leading to an edge misclassification. The middle image of Figure 17 and some examples of Figure 18a,b show correct classification of yellow marking edges. However, the right image of Figure 20 is an example where, even in a same image, some edges due to yellow markings are correctly classified as material change, whereas others are misclassified as shadow edges. This error could be addressed by assuming that the darker region of a yellow marking edge (i.e., the road) is bright enough to not be considered as a shadowed region.

#### 5.2.5. Comparison with Previous Works

The performance of the proposed method is compared with the following five state-of-the-art shadow detection methods:

**Method 1.** The physics-based method in Reference [29] for shadowed road detection exploits intensity thresholding and normalized RGB color space to compute the bluish effect of shadowed road. The normalized blue component *b* and the intensity *I* of shadowed pixels have to satisfy *b* ≥ 1/3 and *I* ≤ *I_road,avg_* – 2*σ_road_*, respectively, where we compute the mean *I_road,avg_* and variance *σ_road_* of the non-shadowed road from the brighter region of the edge under evaluation.

**Method 2.** The physics-based method in Reference [12] exploits the fact that the intensity change between shadowed and non-shadowed surfaces is higher in the red and green components than in the blue. This method uses shadow pixel intensity reduction and albedo ratio test. We adapt the latter to still images using neighboring pixels of regions across the edge under evaluation.

**Method 3.** Using the HSV color space, the color invariance method in Reference [11] assumes that shadow reduces the luminance *v* and saturation *s* components of the surface, whereas the hue *h* varies within a range. We set the threshold values as in Reference [11], i.e., *α*, *β*, *τ_s_*, and *τ_h_* equal to 0.4, 0.6, 0.1, and 0.5, respectively.

**Method 4.** The method in Reference [45] is based on illuminant invariance where an edge is classified as shadow edge if, at a given location, the original image has a strong edge but the illuminant-invariant image has a weak one, or if both images have strong edges but their orientation is different. As in Reference [45], the thresholds *τ_1_*, *τ_2_*, and *τ_3_* are set to 0.4, 0.1, and π/4, respectively, and the image characteristic direction *θ* is computed using the minimum entropy method in Reference [1], obtaining 47°, 54°, and 73° for the Caltech Lane, Kitti Road, and our dataset, respectively.

**Method 5.** The method in Reference [50] is also based on illuminant invariance which determines the illumination spectral direction (ISD) by means of shadowed and non-shadowed road regions. To this end, the method generates two maps of potential shadow pixels and potential lit pixels. Potential shadow pixels are defined as having a low-percent variance (<2%) in each color band, and a color which is roughly neutral but not more blue than a neutral surface is the ISD of sunset. Potential lit pixels are defined as having a low-percent variance (<2%) in each color band, and no color bands are 45% brighter than the other. In addition, the difference between the shadow and lit maps must be at least 0.3 in log(RGB) space in all channels. The ISD computed from the shadow and lit maps must be within a Euclidean distance of 0.1 of the arc on the unit sphere defined by a neutral ISD = (0.577, 0.577, 0.577) and sunset ISD = (0.789, 0.547, 0.299).

Method 1 compares the pixel intensity between candidate shadow pixels and a sample region on the non-shadowed road, whereas Methods 2 and 3 are for image sequences where an initial frame void of shadows is required for comparing the surface properties. Method 5 finds potential shadow and lit maps from a trapezoidal ROI which corresponds approximately to the road surface. Since the aim is to compare the shadow properties exploited by each method, the methods are evaluated under the same conditions for our shadow edge detection strategy. Thus, after extracting the strong edges of the ROI, the darker regions are candidate shadow regions for the five methods, whereas their respective brighter regions are assumed to be the reference non-shadowed regions. For Method 4, we use the strong edges obtained by our classification strategy as edges of the original image.

**Figure 21:**
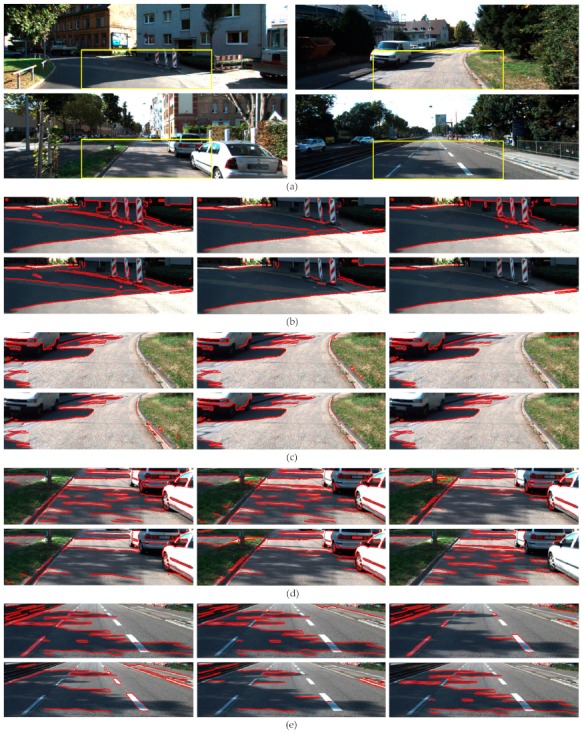
Shadow edge detection results obtained using the five methods. (**a**) ROI overlaid onto incoming images. (**b**–**e**) *Top-left* results of Method 1; *top-center* results of Method 2; *top-right* results of Method 3; *bottom-left* results of Method 4; *bottom-center* results of Method 5; *bottom-right* results of our method.

**Figure 22:**
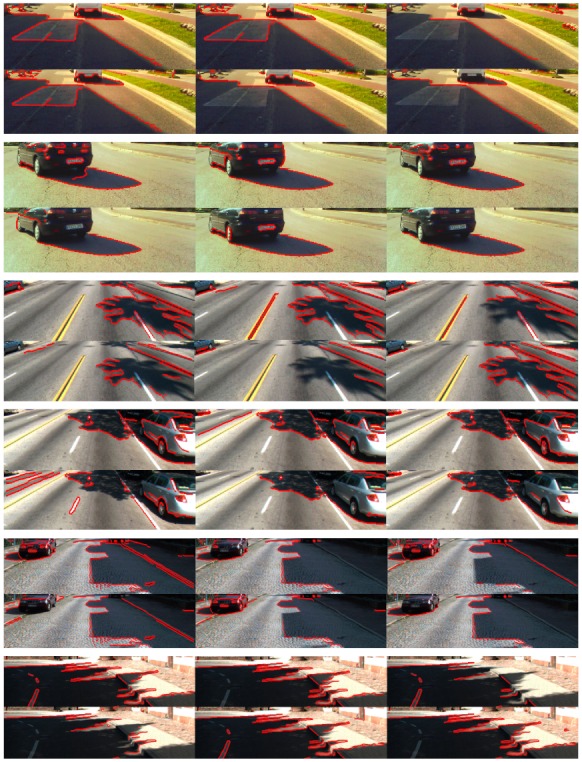
Shadow edge detection results obtained using the five methods. (**First and second scenes***)* ROI of images of our dataset; (**third and fourth scenes***)* ROI of images of the Caltech Lane dataset; (**fifth and sixth scenes***)* ROI of images of the Kitti Road dataset. For each scene: *Top-left* results of Method 1; *top-center* results of Method 2; *top-right* results of Method 3; *bottom-left* results of Method 4; *bottom-center* results of Method 5; *bottom-right* results of our method.

Figure 21 shows the shadow edge detection results obtained by the five methods and ours in four challenging traffic scenes of the Kitti Road dataset. The scenes in Figure 21b,c contain dark shadows caused by the building and parked vehicle, respectively, which generate well-defined shadow edges on the road. The scenes in Figure 21d,e show weak shadows caused by the branches of the trees which generate soft shadow boundaries. The results and comparisons are summarized as follows:

1. Figure 21b,c show that the six methods successfully detect the well-defined shadow edges caused by the building and parked vehicle. The right side of Figure 21b shows that the contour of the thin and weak shadow (penumbra) on the road is also correctly detected by Methods 2 and 4, as well as ours, but not Methods 1, 3, and 5. Similarly, the scene of Figure 21c presents weak shadows on the bottom-left and upper-center regions of the image, whose edges are correctly detected by Methods 1, 2, and 4, as well as ours, but not Methods 3 and 5.

2. Figure 21d,e demonstrate the effectiveness of Methods 1 and 2,as well as ours, in the detection of soft edges separating umbra from penumbra (internal shadow contours) and penumbra from non-shadowed road (external shadow contours), whereas Methods 3–5 miss most of them.

3. Regarding the classification of material-change edges, the four scenes show that the effectiveness of Methods 1–5 is lower than that of our method. Figure 21b shows that Methods 1, 2, and 4 fail to classify the edges due to the asphalt change. Furthermore, they and Methods 3 and 5 misclassify some edges due to material changes in the background of the images of Figure 21b,e. Figure 21c,d show that Methods 1–3 and 5 incorrectly detect bright and material changes on the parked vehicles, whereas Method 4 correctly classifies them in Figure 21c but fails in Figure 21d. An important drawback of Methods 1 and 3–5 is that they easily misclassify edges due to white lane markings on the road (see Figure 21e), which makes them unreliable for lane detection applications. In contrast, the effectiveness of our method in identifying material-change edges can be observed in the correct detection of edges corresponding to asphalt patches, background objects, parked vehicles, and white lane markings. To better show the performance of Methods 1–5 and ours, Figure 22 shows some example results on images of the three datasets. The quantitative results of Methods 1–5 and ours on each of the three datasets are summarized in Table 2. The precision achieved by Methods 1 and 2, as well as ours, is high, with Method 2 being the most effective with a value of 0.911, closely followed by ours with 0.905. The precision of Methods 3–5 is lower, achieving 0.420, 0.637, and 0.594, respectively. With regard to recall, our method achieves 0.884, which indicates high effectiveness in the classification of material-change edges, whereas the recall of Methods 1–5 is lower, achieving values of 0.511, 0.583, 0.442, 0.476, and 0.545, respectively. Finally, our method achieves the highest F-measure (0.894), followed by Method 2 with a value of 0.711. Since the F-measure indicates the global performance of the methods, encompassing both precision and recall, the F-measure achieved by our method demonstrates effectiveness and robustness in classifying both shadow and material-change edges.

It must be said that Methods 1–3 do not focus on detecting shadow edges but on shadow segmentation, coping with umbras where their indicators are significantly higher. On the other hand, the performance of Methods 4 and 5 is highly dependent of the image quality. A higher image quality results in a more accurate illuminant invariant image and better shadow detection results. This is shown in Table 2, as Methods 4 and 5 achieve better results on the Caltech Lane and Kitti roads datasets. It must be said that both Methods 4 and 5 would achieve much more better results using high-quality images with a wide dynamic range captured by calibrated sensors.

## 6. Conclusions

Vision-based driving assistance methods are significantly affected by shadows on the road, which hinder important tasks such as road and lane detections. Additionally, the identification of shadows is not easy since shadow properties may be shared by objects in the scene. The aim of this work was, on the one hand, to find new physical properties to better characterize shadows on the road so as to minimize possible misclassification of objects and non-shadowed image regions as shadows, and, on the other hand, to use the new chrominance properties to design an effective shadow detection method for integration in an onboard road detection system for driver assistance.

We discussed the illumination in outdoor scenes under sunny conditions, which comprise two light sources with different SPDs, i.e., skylight and sunlight, as well as their effect on the road surface. Unlike other methods, when comparing shadowed and non-shadowed regions of the same material surface, we observed the importance of the sunlight contribution to the non-shadowed surface chromaticity, and then derived three new chrominance properties of shadows. Based on the six constraints associated with these properties, we proposed a shadow edge detection method for onboard systems. In as much as onboard systems deal with still images, our method focuses on distinguishing shadow boundaries from material changes by comparing properties of regions across image edges. However, as no prior knowledge of the scene, camera calibration, or spatio-temporal restrictions are required, static background applications can also be addressed.

Tests carried out on different datasets demonstrated the effectiveness of the proposed method in identifying both well-defined and soft shadow edges, achieving precision of 0.905. However, the most remarkable feature of our method is its ability in identifying material-change edges. This demonstrates that the accumulation of shadow feature constraints contributes to a better characterization of shadows by minimizing the possibility of errors in the classification of objects and non-shadowed regions. The proposed method achieved recall and F-measure of 0.884 and 0.894, respectively, showing better performance compared with five state-of-the-art methods.

Although the experiments demonstrated the effectiveness and reliability of our method, there are three limitations, including misclassification of edges due to yellow markings on the road, and missing shadow edges in overexposed and underexposed image regions. The former can be addressed by taking into account the intensity of the candidate shadow region, and the latter could be minimized by using cameras of higher dynamic range.

As future work, we will address the limitations of our method, as well as develop a road detection system for driver assistance.

## Figures and Tables

**Table 1:** Precision (*P*), recall (*R*), and F-measure (*F-m*) indicators.

	Caltech Dataset (100 Images)			Kitti Dataset (100 Images)			Our Dataset (100 Images)			All Datasets (300 Images)		
	*P*	*R*	*F-m*	*P*	*R*	*F-m*	*P*	*R*	*F-m*	*P*	*R*	*F-m*
**Property 1**	0.964	0.791	0.869	0.960	0.727	0.827	0.913	0.672	0.774	0.950	0.730	0.826
**Property 2**	0.968	0.795	0.873	0.963	0.815	0.883	0.931	0.518	0.666	0.959	0.737	0.833
**Property 3**	0.935	0.675	0.784	0.939	0.737	0.826	0.918	0.688	0.787	0.932	0.706	0.804
**Method**	0.901	0.869	0.884	0.926	0.895	0.910	0.888	0.750	0.813	0.905	0.884	0.894

**Table 2:** Precision (*P*), recall (*R*), and F-measure (*F-m*) indicators of the four methods.

	Caltech Dataset (100 Images)			Kitti Dataset (100 Images)			Our Dataset (100 Images)			All Datasets (300 Images)		
	*P*	*R*	*F-m*	*P*	*R*	*F-m*	*P*	*R*	*F-m*	*P*	*R*	*F-m*
**Method 1**	0.827	0.492	0.617	0.870	0.468	0.609	0.822	0.575	0.677	0.839	0.511	0.634
**Method 2**	0.919	0.596	0.723	0.938	0.566	0.706	0.876	0.588	0.704	0.911	0.583	0.711
**Method 3**	0.350	0.426	0.384	0.466	0.440	0.453	0.445	0.461	0.453	0.420	0.442	0.430
**Method 4**	0.646	0.447	0.528	0.724	0.578	0.643	0.541	0.405	0.418	0.637	0.476	0.544
**Method 5**	0.608	0.565	0.585	0.698	0.630	0.662	0.477	0.442	0.458	0.594	0.545	0.568
**Our Method**	0.901	0.869	0.884	0.926	0.895	0.910	0.888	0.750	0.813	0.905	0.884	0.894
